# Transfusion of Resting Platelets Reduces Brain Hemorrhage After Intracerebral Hemorrhage and tPA-Induced Hemorrhage After Cerebral Ischemia

**DOI:** 10.3389/fnins.2019.00338

**Published:** 2019-04-05

**Authors:** Haiyu Luo, Lixiang Wei, Lu Lu, Lijing Kang, Yongliang Cao, Xing Yang, Xiaofei Bai, Wenying Fan, Bing-Qiao Zhao

**Affiliations:** Department of Translational Neuroscience, Jing’an District Centre Hospital of Shanghai, State Key Laboratory of Medical Neurobiology and MOE Frontiers Center for Brain Science, Institutes of Brain Science, Fudan University, Shanghai, China

**Keywords:** cerebral hemorrhage, tPA, blood-brain barrier integrity, platelets, GPVI

## Abstract

**Background:**

Exacerbated blood-brain barrier (BBB) damage is related with tissue plasminogen activator (tPA)-induced brain hemorrhage after stroke. Platelets have long been recognized as the cellular orchestrators of primary haemostasis. Recent studies have demonstrated further that platelets are required for supporting intact mature blood vessels and play a crucial role in maintaining vascular integrity during inflammation. Therefore, we sought to investigate whether platelets could reduce tPA-induced deterioration of cerebrovascular integrity and lead to less hemorrhagic transformation.

**Methods:**

Mice were subjected to models of collagenase-induced intracerebral hemorrhage (ICH) and transient middle cerebral artery (MCA) occlusion. After 2 h of MCA occlusion, tPA (10 mg/kg) was administered as an intravenous bolus injection of 1 mg/kg followed by a 9 mg/kg infusion for 30 min. Immediately after tPA treatment, mice were transfused with platelets. Hemorrhagic volume, infarct size, neurological deficit, tight junction and basal membrane damages, endothelial cell apoptosis, and extravascular accumulation of circulating dextran and IgG, and Evans blue were quantified at 24 h.

**Results:**

Platelet transfusion resulted in a significant decrease in hematoma volume after ICH. In mice after ischemia, tPA administration increased brain hemorrhage transformation and this was reversed by resting but not activated platelets. Consistent with this, we observed that tPA-induced brain hemorrhage was dramatically exacerbated in thrombocytopenic mice. Transfusion of resting platelets ameliorated tPA-induced loss of cerebrovascular integrity and reduced extravascular accumulation of circulating serum proteins and Evans blue, associated with improved neurological functions after ischemia. No changes were found for infarct volume. Inhibition of platelet receptor glycoprotein VI (GPVI) blunted the ability of platelets to attenuate tPA-induced BBB disruption and hemorrhage after ischemia.

**Conclusion:**

Our findings demonstrate the importance of platelets in safeguarding BBB integrity and suggest that transfusion of resting platelets may be useful to improve the safety of tPA thrombolysis in ischemic stroke.

## Introduction

Acute cerebral ischemia is a common form of stroke with high rates of mortality and disability (Roth et al., 2015). Intravenous (IV) injection of the tissue plasminogen activator (tPA) is the only medical therapy approved by the United States Food and Drug Administration (FDA) to treat acute ischemic stroke (Jauch et al., 2013), due to its thrombolytic activity and its ability of restoring circulation to the brain (Hacke et al., 1995). However, thrombolysis induces a markedly increased risk of the intracerebral hemorrhage (ICH), which is fatal in ischemic stroke patient after tPA treatment (Whiteley et al., 2012). However, the appropriate treatment with thrombolysis avoiding hemorrhage in acute ischemic stroke patients has neither been determined. Accumulating clinical and experimental evidence have revealed that tPA exacerbated ischemic endothelial injury and blood-brain barrier (BBB) disruption (Suzuki et al., 2016), which is well recognized the precursor event to hemorrhagic transformation. Thus, improvement of the BBB integrity might be a beneficial strategy for the treatment of thrombolysis-associated ICH.

Platelets are anucleate myeloid blood cells that have a multitude of physiological functions (Ghoshal and Bhattacharyya, 2014). They circulate in blood vessels without forming interactions with non-activated vascular endothelium under normal physiologic conditions (Rasche, 2001). The roles of platelets play in adhering and aggregating at the sites of injured vascular structures, first reported by Bizzozero, have long been recognized as the primary hemostatic function of platelets (Ribatti and Crivellato, 2007). With the expanding breadth of knowledge about platelets, increasing evidence on platelet biology have uncovered a large variety of new functions for platelets. Over the past years, studies show that platelet transfusion prevents hemorrhage in intratumor blood vessels and preserves the vascular integrity independently of forming hemostatic plugs at sites of vascular injury (Ho-Tin-Noe et al., 2008, 2011). More recently, platelets are recognized as central actors of many other pathophysiological processes such as protecting the vasculature against leakage and safeguarding developing vessels, lymphatics, as well as the vascular integrity under inflammatory conditions (Goerge et al., 2008; Lee and Bergmeier, 2016). Meanwhile, it has also been discovered that in thrombocytopenic mice, more hemorrhagic foci are seen around the infarction area after middle cerebral artery occlusion compared with normal mice (Goerge et al., 2008). Platelet has been continuously focused on both experimental stroke and clinical stroke, and there’s increasing evidence suggesting us much more function of platelets should be highlighted besides of the clear aspect on a thromboembolic basis (Mancuso and Santagostino, 2017).

Many academic institutions have developed their own urgent care pathways for managing tPA-associated hemorrhage including the use of platelets (Goldstein et al., 2010). However, there’s few reliable research data available to provide evidence for clinical treatment with platelets for thrombolysis-associated ICH. In this study, we investigated the role of platelets on tPA-induced BBB disruption and hemorrhage after cerebral ischemia. We found that platelet transfusion significantly blocked tPA-associated loss of cerebrovascular integrity, and protected BBB permeability, consequently reduced tPA-induced cerebral hemorrhage.

## Materials and Methods

### Animal Stroke Model

All experimental procedures were approved by the Animal Care and Use Committee of Institutes of Brain Science, Fudan University. All animals used were adult male C57BL/6J mice (Shanghai SLAC Laboratory Animal Co., Shanghai, China) weighing 21 to 28 g at 8 to 10 weeks of age. The ICH model was induced by a striatum injection of 0.025 U collagenase type VII (Sigma-Aldrich, St Louis, MO, United States) in 0.5 μL saline with a 26-gauge needle inserted into the striatum through a burr hole on the skull (Matsushita et al., 2011). After removing the needle, 6 × 10^8^ washed platelets were transfused IV into mice (Gros et al., 2015). Transient focal cerebral ischemia was induced as previously reported (Wang et al., 2013). In brief, mice were subjected to transient occlusion of the right middle cerebral artery (MCAO) with a 7.0 siliconized filament through the external carotid artery. The filament was removed to allow reperfusion after occlusion for 45 min. Anesthesia was induced with 1 to 1.5% isoflurane in a mixture of 30% oxygen and 70% nitrogen. During the operations, the body temperature was maintained at 37 ± 0.5°C using a heating pad. Successful MCA occlusions and reperfusion were confirmed by Laser Doppler flowmetry (Perimed, Stockholm, Sweden), and only animals with a >70% reduction in cerebral blood flow after MCAO and a >50% of baseline cerebral blood flow after filament withdrawal were included in this study. At 2 h after MCAO, tPA (10 mg/kg, Actilyse, Mannheim, Germany) or PBS was administered as an intravenous bolus injection of 1 mg/kg followed by a 9 mg/kg infusion for 30 min with a syringe infusion pump (World Precision Instruments, Sarasota, FL, United States). D-glucose (6 mL/kg at 50% wt/vol, Sigma-Aldrich) was used to increase the extent of cerebral hemorrhage by an intraperitoneal injection 15 min before MCAO as described previously (Wang et al., 2013). Mice were transfused with 6 × 10^8^ washed platelets immediately after tPA treatment. Mice that died after MCAO surgery were excluded from the end-point analyses. The mortality rates in each study were as follows: among the collagenase-induced ICH mice (*n* = 7 per group), the mortality rate was 12.5% (1/8) in ICH group, 0% (0/7) in ICH + platelets or tyrode buffer group; among the ischemic mice, the mortality rate was 10.9% (5/46) in vehicle group, 22.6% (12/53) in tPA group, 14.9% (10/67) in tPA + platelets group, 24.1% (13/54) in tPA + tyrode buffer group, 20% (2/10) in tPA + activated platelets group, 20% (4/20) in tPA + IgG group, 42.8% (12/28) in tPA + anti-GPIbα group, 23.8% (5/21) in tPA + JAQ1-platelets group.

### Platelet Preparation

Washed platelets were prepared from WT mice as described (Cazenave et al., 2004). Donor mice were anesthetized by isoflurane, and whole blood was collected from the retroorbital venous plexus and sampled in acid-citrate-dextrose (ACD: 2.5% trisodium citrate, 2% D-glucose and 1.5% citric acid) [at 1 (ACD): 9 (blood) ratio]. To obtain washed platelets, the sampled blood was first centrifuged at 800 rpm, 4 min, then the platelet-rich fraction and part of the red blood cells were collected with supplement of 1 μL prostacyclin (PGI_2_, 1 mg/mL, Sigma-Aldrich) and further centrifuged at 1200 rpm, 4 min. The platelet-rich plasma (PRP) was collected and added with 1 μL PGI_2_. After centrifugation (2600 rpm, 4 min), the isolated platelets were washed twice with HEPES-Tyrode’s buffer and resuspended to a concentration of 6 × 10^9^ platelets/mL.

### Platelet Depletion and Platelet Activation

Platelet depletion was induced by an intravenous injection of 1 μg/g bodyweight of the platelet-depleting antibody (GPIbα antibody; Emfret Analytics, Würzburg, Germany) or matched non-immune isotype antibody (Emfret Analytics) to mice immediately after tPA treatment. Platelet counts were evaluated by flow cytometry. Platelets in the presence of 2 mmol/L ethylenediaminetetraacetic acid (EDTA) to avoid aggregation were stimulated with human thrombin (1 units/mL, Sigma-Aldrich), an activator of platelets for 10 min at 37°C. Hirudin (2 units/mL, Sigma-Aldrich), a specific inhibitor of thrombin was added to stop the reaction (Ho-Tin-Noe et al., 2008). Eight mice were evaluated per group for measurements of hemorrhagic volume, BBB permeability and flow cytometry.

### Flow Cytometry

Platelet-rich plasma was prepared by centrifugation of anticoagulated blood as previously described (Vaiyapuri et al., 2012). For flow cytometry, 10 μL of this suspension sample was incubated with 1 μL of fluorescein isothiocyanate (FITC) conjugated CD61 MoAb (BD Pharmingen, San Diego, CA). Immediately afterward, 39 μL of PBS was added with gentle mixing. After 30 min of incubation at room temperature in the dark, 450 μL of PBS was added and the samples were analyzed on a FACSCalibur flow cytometer (BD Bioscience) using CellQuest (BD Bioscience) and FlowJo (TreeStar) software (Arroyo et al., 2001).

### Inhibitor Studies

To inhibit GPVI function, washed platelets were incubated with the inhibitory antibody JAQ1 (100 μg/mL, Emfret Analytics) *in vitro*, after 15 min, the unbound JAQ1 antibody was washed away (Boulaftali et al., 2013). The JAQ1-pretreated platelets were transfused into mice (*n* = 8 per group) as described above.

### Quantification of Cerebral Hemorrhage

Intracerebral hemorrhage was quantified with a spectrophotometric assay as previously described (Wang et al., 2013). Mice (*n* = 8 per group) were anesthetized and perfused transcardially with ice-cold PBS at 24 h after MCAO. The ischemic hemisphere of each mouse was homogenized and centrifuged at 13,000 rpm for 30 min with Drabkin reagent (500 μL, Sigma-Aldrich). Optical density of supernatants was measured at 540 nm by spectrophotometry (Thermo Fisher Scientific BioMate 3S; Thermo Fisher Scientific, Waltham, MA, United States). A reference curve was generated by blending different known volumes of fresh mouse blood into normal brain samples. Hemorrhage volume was expressed in equivalent units by comparison with this reference curve.

### BBB Permeability Evaluation

At 23 h after MCAO (*n* = 8 per group), Evans blue dye (4% in PBS, 4 mL/kg, Sigma-Aldrich) injections were given intravenously and allowed to circulate for 1 h. The ischemic hemispheres were weighted and placed in formamide for 72 h in the dark. The amount of extravasated Evans blue dye was evaluated by spectrophotometry (Thermo Fisher Scientific BioMate 3S; Thermo Fisher Scientific) at 620 nm (Cai et al., 2015).

### Detection of Extravascular Dextran and IgG

A tracer FITC-labeled dextran was used to estimate the *in vivo* BBB permeability as previously described (Xu et al., 2017). Briefly, vascular leakage was analyzed after IV injection of 40 kDa FITC-dextran (50 μL of 100 mg/mL, Sigma-Aldrich) circulating for 2 min. The brains were quickly removed and postfixed in 4% paraformaldehyde at 4°C overnight, and then immersed 48 h in 30% sucrose in PBS. 50 μm-thick coronal brain sections were immunostained with goat anti-CD31 (AF3628, R&D Systems, Minneapolis, MN, United States) and incubated with Alexa Fluor 594-conjugated donkey anti-goat IgG (Invitrogen). The images of extravascular dextran fluorescence were processed by ImageJ software and the dextran leakage was quantified as a percentage of dextran^+^ area. Extravascular IgG was measured by incubating cerebral tissue sections with a combination of antibodies of goat anti-CD31 (AF3628, R&D Systems), Alexa Fluor 488-conjugated donkey anti-mouse IgG and Alexa Fluor 594 donkey anti-goat IgG (Invitrogen) (Xu et al., 2017). The images were digitized using a 40× objective in the peri-infarct area and the immunofluorescent intensity of extravascular IgG was quantified using the ImageJ Integrated Density analysis measurement tool. Five mice were used in each group.

### Measurement of Infract Volume

Mice (*n* = 5 per group) were sacrificed by overdose of chloral hydrate and the brains were cut into 1-mm thick coronal slices using a rodent brain matrix. Eight selected sections were stained for 30 min in a 2% solution of triphenyl-2, 3, 4-tetrazolium-chloride (TTC, Sigma-Aldrich). Sections were digitalized and the volume of ischemic brain injury was measured blindly using the ImageJ software (Fan et al., 2016).

### Western Blotting

The ischemic hemispheric brain tissues and matching tissues from sham-operated mice (*n* = 5 per group) were removed and used for western blot as previously described (Wang et al., 2013). The primary antibodies were: rabbit anti-collagen IV (ab6586, Abcam, Cambridge, MA, United States), rabbit anti-ZO-1 (617300, Invitrogen, Camarillo, CA, United States), rabbit anti-occludin (ab167161, Abcam), and rabbit anti-β-actin (4970, Cell Signaling Technology, Beverly, MA, United States).

### Immunohistochemistry

After transcardial perfusion/fixation with ice-cold 4% paraformaldehyde and immersed in 30% sucrose in PBS at 4°C overnight, the brain tissues (*n* = 5 per group) were cut into 20 μm sections. Immunohistochemistry was performed as previously described (Xu et al., 2017). The primary antibodies used were: goat anti-CD31 (AF3628, R&D Systems, Minneapolis, MN, United States), rabbit anti-collagen IV (ab19808, Abcam), rabbit anti-ZO-1 (617300, Invitrogen, Camarillo, CA, United States), rabbit anti-occludin (ab404700, Abcam), rabbit anti-fibrinogen (AP00766PU-N, Acris Antibodies, CA, United States). The secondary antibodies used were: Alexa Fluor 594-conjugated donkey anti-goat IgG, Alexa Fluor 488-conjugated donkey anti-rabbit IgG (all from Invitrogen). Images were obtained using an Olympus BX 51 microscope and an Olympus FV1000 confocal microscope. For quantification of the positive cells, 6 randomly selected non-overlapping images (40× objective) in the peri-infarct area were digitized and analyzed using ImageJ software.

### Immunofluorescence Double Staining for CD31 and TUNEL

TUNEL staining was performed on brain cryosections according to the manufacturer’s manual of *in situ* cell death detection kit (Roche Diagnostics, Mannheim, Germany) followed by CD31 immunofluorescent staining as described (Bruns et al., 2000). We determined the percentage of TUNEL-positive endothelial cells in the peri-infarct area using six different microscopic fields per section at 400× magnification and five mice were evaluated per group.

### Behavioral Measurements

At 24 h after MCAO, mice (*n* = 10 per group) were examined and scored blindly using the modified Neurological Severity Scores (mNSS) (Chen et al., 2001). The mNSS is a composite of motor, sensory, beam balance and reflex tests by a grading scale of 0 (normal score) to 18 (maximal deficit score).

### Statistical Analysis

Data are expressed as the mean ± SD and were assessed with one-way analysis of variance after the Bonferroni multiple comparison test. When two groups were compared, values were analyzed using unpaired 2-tailed Student *t-test*. Behavior data were analyzed using Kruskal–Wallis test followed by the Dunn multiple comparison test. Differences were considered statistically significant with *p-values* <0.05.

## Results

### Transfusion of Resting Platelets Reduces Brain Hemorrhage After ICH and Cerebral Ischemia

**FIGURE 1:**
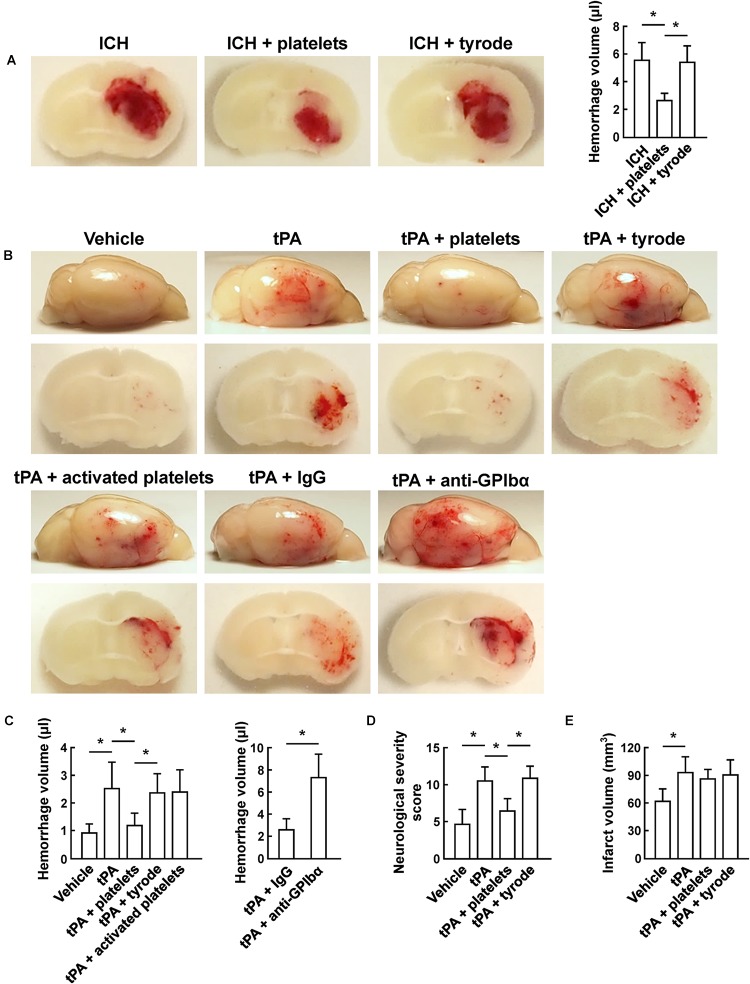
Transfusion of resting platelets reduced hemorrhage volume after ICH and ischemic stroke. **(A)** Representative images of coronal sections and quantification of brain hemorrhage 24 h after collagenase-induced ICH in mice injected with vehicle, platelets and tyrode. *n* = 7 per group. **(B)** Representative images of the dorsal surface and coronal sections show brain hemorrhage 24 h after cerebral ischemia in mice treated with vehicle, tPA, tPA + resting platelets, tPA + tyrode buffer, tPA + activated platelets, tPA + control IgG, or tPA + platelet-depleting IgG (GPIbα). **(C)** Quantification of cerebral hemorrhage for each group. *n* = 8 per group. **(D)** Neurological severity score (NSS) 24 h after cerebral ischemia in mice treated with vehicle, tPA, tPA + resting platelets, or tPA + tyrode buffer. *n* = 10 per group. **(E)** Quantitative analysis of infarction volume 24 h after cerebral ischemia in mice treated with vehicle, tPA, tPA + resting platelets, or tPA + tyrode buffer. *n* = 5 per group. Values are mean ± SD. ^∗^*P* < 0.05.

Using the collagenase-induced ICH model, we found that transfusion of resting platelets significantly reduced cerebral hematoma volume compared with vehicle treatment at 24 h after ICH (Figure 1A). To investigate the role of platelets on tPA-mediated hemorrhage after ischemic stroke, we subjected mice to 45 min of focal cerebral ischemia using the MCAO model. As reported, infusion of tPA 2 h after MCAO resulted in a significant increase in hemorrhage (Wang et al., 2013). Treatment with resting platelets successfully blocked tPA-induced hemorrhage, whereas thrombin-activated (degranulated) platelets failed to reduce the hemorrhage (Figure 1B,C). These data suggest that mediators released from platelet granules might be critical for preventing tPA-induced hemorrhage. A recent study also shows that platelet secretion is associated with cerebral hemorrhage after ischemia (Deppermann et al., 2017). Moreover, in our study, combination treatment with resting platelets and tPA did not affect infarct volume (Figure 1E) and intravascular fibrin deposits (Supplementary Figure S1B) compared with the tPA alone group. Previous study showed that platelet depletion by anti-GPIbα antibody alone increased the hemorrhage compared with the mice with normal platelet count after MCAO (Zhao et al., 2009). To further confirm the role of platelets on tPA-induced hemorrhage, we induced thrombocytopenia in mice by platelet depletion using an anti-GPIbα antibody (Supplementary Figure S1A). These experiments showed that treatment with tPA led to more severe hemorrhage in thrombocytopenic mice compared with mice with normal platelet counts (Figure 1B,C). In addition, the tPA-treated mice transfused with platelets had better neurological scores than those without platelet transfusion (Figure 1D).

### Resting Platelets Block tPA-Induced Increase in BBB Permeability and Preserve Vascular Integrity After Cerebral Ischemia

**FIGURE 2:**
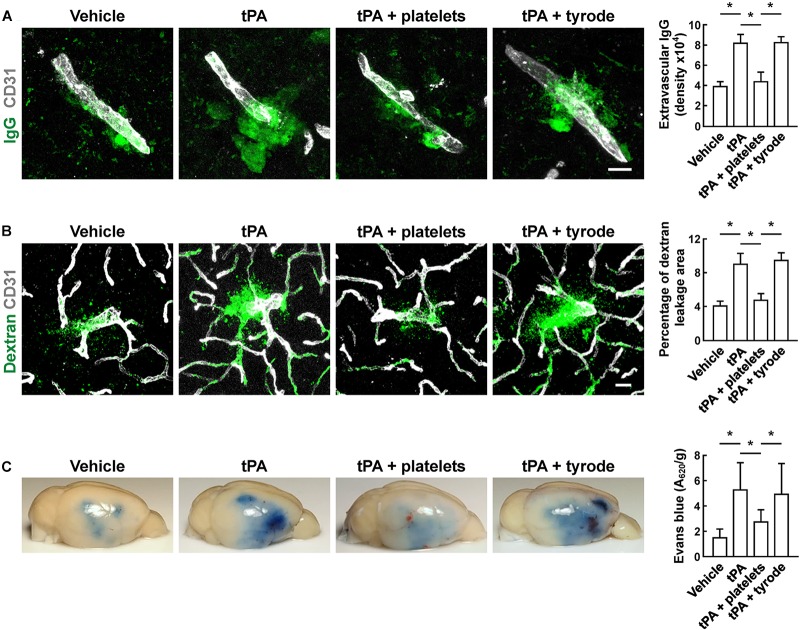
Transfusion of resting platelets reduced tPA-induced BBB disruption after cerebral ischemia. **(A)** Representative images and quantitative analysis of IgG leakage 24 h after cerebral ischemia in mice treated with vehicle, tPA, tPA + resting platelets, or tPA + tyrode buffer. Bar = 10 μm. *n* = 5 per group. **(B)** Representative images and quantitative analysis of extravascular dextran fluorescence in mice treated with vehicle, tPA, tPA + resting platelets, or tPA + tyrode buffer. Bar = 20 μm. At 24 h after stroke, mice were given an intravascular injection of 40,000 Da FITC-labeled dextran, and brain sections were stained with CD31. *n* = 5 per group. **(C)** Representative photographs and quantification of Evans blue dye extravasation 24 h after cerebral ischemia. *n* = 8 per group. Values are mean ± SD. ^∗^*P* < 0.05.

**FIGURE 3:**
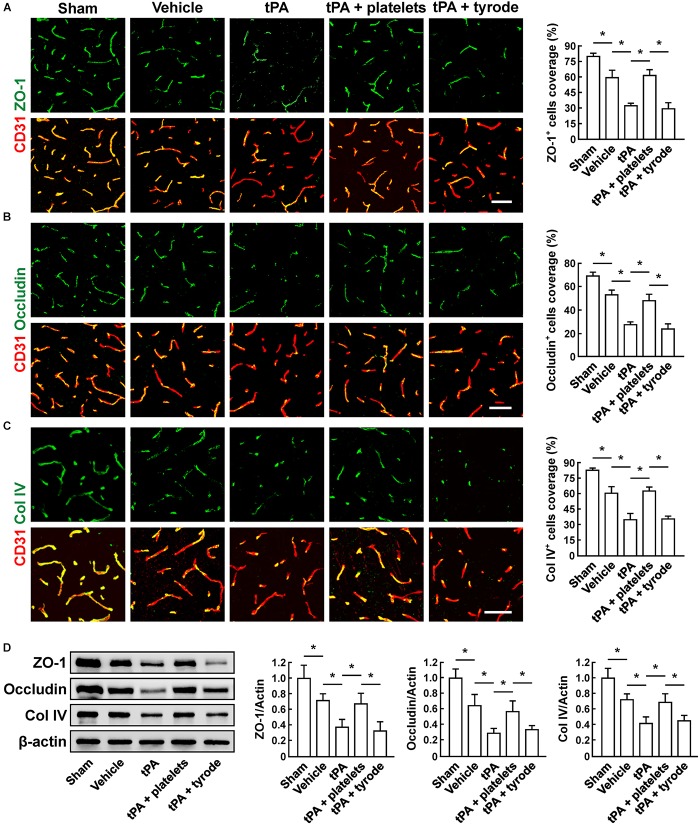
Transfusion of resting platelets ameliorated tPA-induced loss of cerebrovascular integrity after cerebral ischemia. **(A–C)** Representative images and quantification of tight junction protein zonula occludens (ZO-1) **(A)**, occludin **(B)**, and basal membrane protein collagen IV (Col IV) **(C)** coverage on CD31+ microvessels in sham-operated mice and mice treated with vehicle, tPA, tPA + resting platelets, or tPA + tyrode buffer 24 h after cerebral ischemia. Bar = 60 μm. *n* = 5 per group. **(D)** Representative immunoblots and quantification of ZO-1, occludin, and collagen IV in the brain extracts from sham-operated mice and mice treated with vehicle, tPA, tPA + resting platelets, or tPA + tyrode buffer after cerebral ischemia. *n* = 5 per group. Values are mean ± SD. ^∗^*P* < 0.05.

**FIGURE 4:**
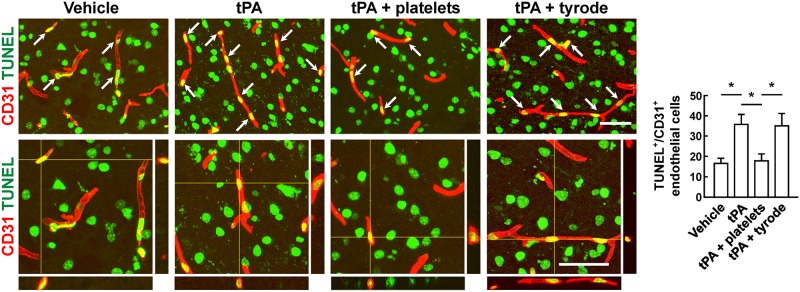
Transfusion of resting platelets reduced tPA-induced loss of vascular endothelial cells after cerebral ischemia. Representative photomicrographs and quantification of TUNEL+/CD31+ endothelial cells in mice treated with vehicle, tPA, tPA + resting platelets, or tPA + tyrode buffer after cerebral ischemia. Bar = 60 μm. *n* = 5 per group. Values are mean ± SD. ^∗^*P* < 0.05.

To evaluate the BBB damage, we quantified the perivascular IgG deposits, leakage of dextran (MW = 40,000 Da), and Evans blue infiltration to the brain parenchyma. The results showed that tPA exacerbated the disruption of the BBB, but these effects were reversed by platelet transfusion (Figure 2A–C), which revealed that resting platelets resulted in a protective role of BBB function. Furthermore, we investigated the role of resting platelets on tPA-mediated degradation of BBB components after stroke. The dual staining for the BBB tight junction proteins including ZO-1 and occludin, each combined with CD31, indicated significant increases in ZO-1^+^ coverage and occludin^+^ coverage in platelets-treated mice (Figure 3A,B). Mice with platelet transfusion also exhibited increase in the major vascular basal membrane protein collagen IV, as shown by immunostaining of brain microvessels (Figure 3C). Together with these findings, western blot analysis showed that treatment of mice with platelets remarkably preserved the loss of these three proteins (Figure 3D). We next determined the effects of platelets on vascular injury based on the data above using double staining of CD31 (endothelial cells) and TUNEL (apoptotic cells). tPA critically enhanced endothelial cell apoptosis after cerebral ischemia, whereas resting platelets substantially diminished tPA-mediated vascular damage (Figure 4).

### Resting Platelets Prevent tPA-Induced Hemorrhage and Reduce BBB Damage via GLYCOPROTEIN VI (GPVI)-Dependent Manner

**FIGURE 5:**
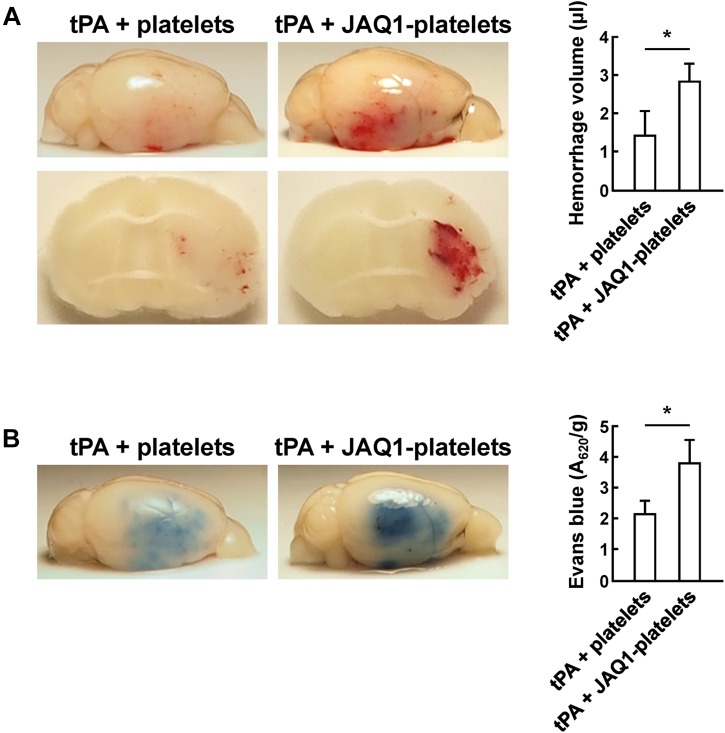
Platelets reduced tPA-induced hemorrhage and BBB disruption in a GPVI-dependent manner. **(A,B)** Representative images and quantification of brain hemorrhage **(A)** and Evans blue dye extravasation **(B)** 24 h after cerebral ischemia in mice treated with tPA + platelets, or tPA + JAQ1-platelets. Immediately after tPA treatment, mice were transfused with platelets or anti-GPVI antibody (JAQ1)-pretreated platelets. *n* = 8 per group. Values are mean ± SD. ^∗^*P* < 0.05.

Glycoprotein VI is the major collagen receptor mediating primary adhesion of platelets to the vascular wall (Nieswandt and Watson, 2003). Recent evidence exposed that the vascular protective role of platelets was independent of their ability to form thrombi (Goerge et al., 2008; Ho-Tin-Noe et al., 2011), and inhibition of GPVI partially abolished the protective function of platelets on vascular integrity during inflammation (Boulaftali et al., 2013; Gros et al., 2015). We speculated that whether GPVI act primarily in the maintenance of cerebrovascular integrity by preventing hemorrhage and regulating BBB permeability after cerebral ischemia. We first incubated resting platelets *in vitro* with a blocking antibody JAQ1 against GPVI before platelet transfusion. Compared to controls, JAQ1-pretreated platelets did not lessen hemorrhage volume (Figure 5A), and the mice with platelets lacking GPVI accumulated significant Evans blue extravasation (Figure 5B). Inhibition of platelet receptor GPVI exhibited no vasculoprotective function, and impaired the ability of resting platelets to reduce tPA-induced hemorrhage and maintain vascular permeability after cerebral ischemia. Our results indicated that the protective effect of platelets in supporting vascular integrity was closely involved in GPVI-dependent signal transduction pathway.

## Discussion

In this study, we showed that resting platelets significantly reduced tPA-mediated loss of cerebrovascular integrity and BBB breakdown, thereby preventing tPA-induced cerebral hemorrhage and improving functional outcomes after cerebral ischemia.

It is well known that platelet adhesion, activation and aggregation are key to thrombosis, which may further lead to thrombotic diseases (Ruggeri, 2002; Mackman, 2008). Historically, antiplatelet therapy for acute stroke was frequently utilized to inhibit platelet activation and aggregation (Bednar and Gross, 1999), which has also been widely combined with thrombolytics in secondary stroke prevention (Luo et al., 2016). However, antiplatelet therapy has been forcefully proven to induce a higher risk of systemic bleeding and intracranial hemorrhage (Serebruany et al., 2004).

In our study, we demonstrated that platelets could effectively prohibit the collagenase-induced ICH in mice. Consistent with our result above, it has been shown that in some clinical studies, platelet transfusion leads to smaller final hemorrhage size for patients with ICH, which supports a potential treatment of platelet transfusion for ICH (Naidech et al., 2012). Given the evidence that platelets are shown to support vascular development and remodeling, safeguard the lymphatic vessels, secure inflammatory vessels and prevent bleeding at the sites of inflamed organs and tumors (Goerge et al., 2008; Ho-Tin-Noe et al., 2011). We hypothesize that platelet transfusion could protect brain microvessels integrity, further preventing tPA-mediated cerebral hemorrhage. Indeed, our findings provided evidence that platelets played an important role in the maintenance of cerebral vascular integrity, which distinguished from the previous thrombotic propensity of platelets in stroke. In our research, we showed that administration with resting platelets decreased the hemorrhage in tPA-treated mice as well as remarkably lowered neurological scores without affecting the infarction volume and intravascular fibrin deposits. However, the life span of transfused platelet in the body is more than 3 days (Odell and McDonald, 1961). To exclude the possibility that transfusion of resting platelets might increase the risk of secondary microvascular thrombosis, further study would be important to examine 3- or 7-day survival rate and neurological scores after platelet transfusion. Notably, after we activated platelets, they lost their ability to prevent bleeding, indicating that prevention of cerebral hemorrhage by platelets might be dependent upon the localized platelet secretions rather than platelet aggregation. Combination treatment with resting platelets and tPA exhibited significant protection of BBB critical components degradation, and inhibition of BBB disruption as well as reduction of vascular impairment.

The platelet receptor for collagen, GPVI, is believed to be an active participant in preventing excessive blood loss after injury. A previous study found that mice with GPVI deficiency showed defect in hemostasis, as the same results also been confirmed in patients with impaired GPVI expression (Gruner et al., 2004; Hermans et al., 2009). In recent years, GPVI has been found to be pivotal in platelet-endothelium interaction targeting in inflammatory diseases independent of hemostasis and thrombosis, and inhibition of GPVI partially blocks the ability of platelets to prevent inflammatory bleeding (Boulaftali et al., 2013; Gros et al., 2015). Consistently, in acute cerebral ischemia, we demonstrated that inhibition of GPVI impaired the ability of platelets to prevent cerebral hemorrhage. Moreover, platelets deficient in GPVI also showed a defect in blockage of vascular leakage. Together, these data suggest that platelets secure vascular integrity at sites of damaged cerebrovascular maybe mediate via GPVI, and possibly relies on the released platelet granule contents.

## Conclusion

In conclusion, we found that resting platelets maintained microvascular integrity, and decreased tPA-associated hemorrhage in acute ischemic stroke. Our data suggest that platelet transfusion would potentially increase the safety of tPA thrombolytic therapy.

## Ethics Statement

This study was carried out in accordance with the recommendations of the Animal Care and Use Committee of Institutes of Brain Science, Fudan University. The protocol was approved by the Animal Care and Use Committee of Institutes of Brain Science, Fudan University.

## Author Contributions

HL, LW, LL, LK, YC, XY, and XB performed the experiments and analyzed the data. HL, WF, and B-QZ designed the study and wrote the manuscript. All authors read and approved the final manuscript.

## Conflict of Interest Statement

The authors declare that the research was conducted in the absence of any commercial or financial relationships that could be construed as a potential conflict of interest.

## Supplementary Material

FIGURE S1 **(A)** Thrombocytopenia was achieved successfully by injection of an anti-mouse GPIbα antibody. Representative density plots of platelets defined by CD61-positivity and sideward light scatter (SSC) characteristics. CD61-FITC was used to stain platelets, expressed as mean fluorescence intensity values (MFI; relative linear units scaled from 0 to 10^4^), platelet counts expressed as percentage relative to control (100%) were measured with flow cytometry. *n* = 8 per group. Unpaired 2-tailed Student *t-test*. **(B)** Representative confocal microscopy images and quantitative analysis of fibrin deposits in microvessels from mice treated with vehicle, tPA, tPA + resting platelets, or tPA + tyrode buffer 24 h after cerebral ischemia. Bar = 60 μm. *n* = 5 per group. Values are mean ± SD. ^∗^*P* < 0.05.
