# Supplementary figures and images for: Transfusion of Resting Platelets Reduces Brain Hemorrhage After Intracerebral Hemorrhage and tPA-Induced Hemorrhage After Cerebral Ischemia
Source: Front Neurosci. 2019 Apr 5;13:338. doi: 10.3389/fnins.2019.00338 (PMC6460946; doi:10.3389/fnins.2019.00338)

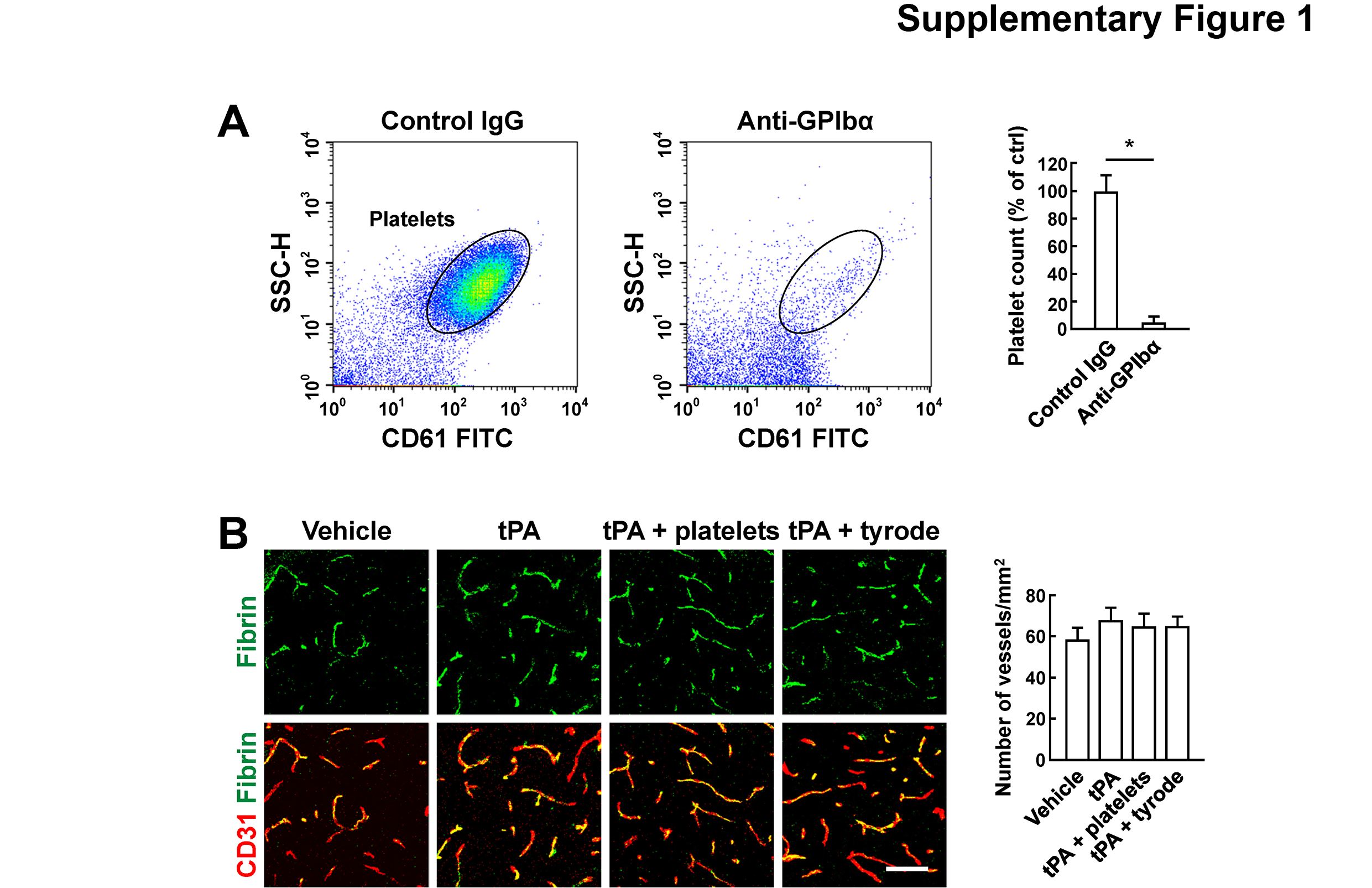

Supplement: FIGURE S1 — (A) Thrombocytopenia was achieved successfully by injection of an anti-mouse GPIbα antibody. Representative density plots of platelets defined by CD61-positivity and sideward light scatter (SSC) characteristics. CD61-FITC was used to stain platelets, expressed as mean fluorescence intensity values (MFI; relative linear units scaled from 0 to 104), platelet counts expressed as percentage relative to control (100%) were measured with flow cytometry. n = 8 per group. Unpaired 2-tailed Student t-test. (B) Representative confocal microscopy images and quantitative analysis of fibrin deposits in microvessels from mice treated with vehicle, tPA, tPA + resting platelets, or tPA + tyrode buffer 24 h after cerebral ischemia. Bar = 60 μm. n = 5 per group. Values are mean ± SD. ∗P < 0.05. [file Image_1.TIF]
